# Feedback Circuit among INK4 Tumor Suppressors Constrains Human Glioblastoma Development

**DOI:** 10.1016/j.ccr.2008.02.010

**Published:** 2008-04-08

**Authors:** Ruprecht Wiedemeyer, Cameron Brennan, Timothy P. Heffernan, Yonghong Xiao, John Mahoney, Alexei Protopopov, Hongwu Zheng, Graham Bignell, Frank Furnari, Webster K. Cavenee, William C. Hahn, Koichi Ichimura, V. Peter Collins, Gerald C. Chu, Michael R. Stratton, Keith L. Ligon, P. Andrew Futreal, Lynda Chin

**Affiliations:** 1 Department of Medical Oncology, Dana-Farber Cancer Institute and Harvard Medical School, Boston, MA 02115, USA; 2 Center for Applied Cancer Science, Belfer Institute for Innovative Cancer Science, Dana-Farber Cancer Institute, Boston, MA 02115, USA; 3 Center for Cancer Genome Discovery, Dana-Farber Cancer Institute, Boston, MA 02115, USA; 4 Center for Molecular Oncologic Pathology, Dana-Farber Cancer Institute, Boston, MA 02115, USA; 5 Department of Neurosurgery, Memorial Sloan-Kettering Cancer Center, New York, NY 10065, USA; 6 Department of Neurosurgery, Weill-Cornell Medical College, New York, NY 10065, USA; 7 Cancer Genome Project, Wellcome Trust Sanger Institute, Wellcome Trust Genome Campus, Hinxton, Cambridge CB10 1SA, UK; 8 Ludwig Institute for Cancer Research, University of California, San Diego, La Jolla, CA 92093, USA; 9 Broad Institute of Harvard and MIT, Cambridge, MA 02142, USA; 10 Department of Pathology, University of Cambridge, Cambridge CB2 1QP, UK; 11 Institute of Cancer Research, Sutton, Surrey SM2 5NG, UK; 12 Department of Pathology, Brigham and Women's Hospital and Harvard Medical School, Boston, MA 02115, USA; 13 Department of Dermatology, Brigham and Women's Hospital and Harvard Medical School, Boston, MA 02115, USA; 14 Department of Medicine, Brigham and Women's Hospital and Harvard Medical School, Boston, MA 02115, USA

**Keywords:** CELLCYCLE

## Abstract

We have developed a nonheuristic genome topography scan (GTS) algorithm to characterize the patterns of genomic alterations in human glioblastoma (GBM), identifying frequent *p18^INK4C^* and *p16^INK4A^* codeletion. Functional reconstitution of p18^INK4C^ in GBM cells null for both *p16^INK4A^* and *p18^INK4C^* resulted in impaired cell-cycle progression and tumorigenic potential. Conversely, RNAi-mediated depletion of *p18^INK4C^* in *p16^INK4A^*-deficient primary astrocytes or established GBM cells enhanced tumorigenicity in vitro and in vivo. Furthermore, acute suppression of *p16^INK4A^* in primary astrocytes induced a concomitant increase in *p18^INK4C^*. Together, these findings uncover a feedback regulatory circuit in the astrocytic lineage and demonstrate a bona fide tumor suppressor role for p18^INK4C^ in human GBM wherein it functions cooperatively with other INK4 family members to constrain inappropriate proliferation.

## SIGNIFICANCE

**Understanding the molecular basis of human cancer and, thereby, targeting the genetic defects in a rational manner requires a comprehensive knowledge of not only the driving pathogenetic lesions but also their interactions. Using a newly developed algorithm that enables the analysis of copy number profiles based on focality, amplitude, and recurrence of the genomic events, we uncovered a codeletion pattern among closely related INK genes in the GBM oncogenome, challenging the prevailing single-hit model of RB pathway inactivation. Elucidation of the molecular basis underlying this codeletion pattern revealed a backup tumor suppressor role for p18^INK4C^ in the setting of *p16^INK4A^* deletion, thus expanding our understanding of human GBM and compensation among INK family members in human tumors.**

## Introduction

GBM, the most common primary brain tumor in adults, is neurologically destructive and maintains dismal responses to virtually all therapeutic modalities. The pathobiology of GBM is characterized by rapid proliferation as well as widespread invasion, robust angiogenesis, apoptosis resistance, and florid necrosis (Furnari et al., 2007). On the molecular level, GBM is characterized by coactivation of receptor tyrosine kinases (Huang et al., 2007; Stommel et al., 2007), activation of PI3K-AKT signaling (Li et al., 1997a; Stambolic et al., 1998), and loss of p53 and RB tumor suppressor pathway function (Furnari et al., 2007).

Disruption of the RB pathway appears to be an obligate event in tumorigenesis and is achieved primarily through either deletion/mutation of *RB*, amplification of the G1 cyclin-dependent kinases 4 or 6 (*CDK4/6*), or deletion/silencing of the G1 CDK inhibitor (CKI), *CDKN2A* (*p16^INK4A^*). In individual human tumor specimens, these principal components of the pathway—RB-CDK4/6-p16^INK4A^—are reported to be targeted in a mutually exclusive manner (Sherr and McCormick, 2002) consistent with their overlapping functions in regulating the G1-S transition of the cell cycle (Massague, 2004). However, recent data in the mouse have challenged this notion of exclusivity. Sharpless and colleagues reported that combined germline nullizygosity for *p16^Ink4a^* and its related family member *p18^Ink4c^* facilitated the development of pituitary tumors in the mouse (Ramsey et al., 2007). Berns and colleagues showed enhanced tumorigenicity and expanded tumor spectrum in mice null for *p15^Ink4b^* and *p16^Ink4a^/Arf*, compared to *p16^Ink4a^/Arf* alone (Krimpenfort et al., 2007). While these genetic data in the mouse have suggested cooperation and/or compensation among members of the Ink4 family of CKI, the relevance to human cancers of these findings in the mouse has not been established.

The recalcitrant nature of GBM and emerging evidence for genes and genomic loci governing response to both targeted and conventional therapy (Cahill et al., 2007; Hunter et al., 2003; Mellinghoff et al., 2005) have motivated efforts to more fully define the genetics of GBM. Previous conventional and array-based comparative genomic hybridization (array-CGH) profiles have revealed numerous recurrent regional copy number aberrations (CNAs) in GBM (Kotliarov et al., 2006; Nigro et al., 2005; Ichimura et al., 2006; Korshunov et al., 2006; Lee et al., 2006; Liu et al., 2006; Mulholland et al., 2006; Rossi et al., 2005). The large numbers of CNAs detected by array-CGH and the resulting long lists of resident genes have highlighted the need for a systematic objective prioritization approach designed to separate true CNA target genes from bystanders.

Here, we report the development of a nonheuristic genome topography scan (GTS) algorithm to define and rank genomic regions exhibiting significant CNAs based on genome-wide array-CGH profiles of primary GBM samples and cell lines. In addition to the expected GBM signature alterations, GTS identified many CNAs not previously implicated in GBM development, uncovered genomic codeletion of two highly related G1 CKIs, *p18^INK4C^* and *p16^INK4A^*, in human GBM tumors and cell lines, leading to discovery and validation of a previously unappreciated cell-cycle regulatory circuit in the astrocytic lineage.

## Results

### GTS Defines Regions of Interest Encompassing Focal and Recurrent CNAs

To identify novel GBM-relevant genes, we performed high-resolution, oligo-based array-CGH profiling to determine the genome-wide CNA patterns of 28 pathologically verified primary Grade IV glioma (GBM) specimens and 18 established glioma cell lines (Table S1 available online). Using a rule-based algorithm to define minimal common regions (MCRs) of CNAs based on amplitude, width, and recurrence of CNAs (Aguirre et al., 2004; Carrasco et al., 2006; Tonon et al., 2005), we readily identified the signature genomic events known previously for GBM (e.g., *EGFR* amplification or *CDKN2A* deletion) as well as many previously uncharacterized alterations (Table S2). In view of the large number of CNAs and the complexity of overlap across samples, we developed a nonheuristic methodology, genome topography scanning (GTS), to more rigorously define and rank genomic regions based on the amplitude, width, and recurrence of a CNA.

**Figure 1:**
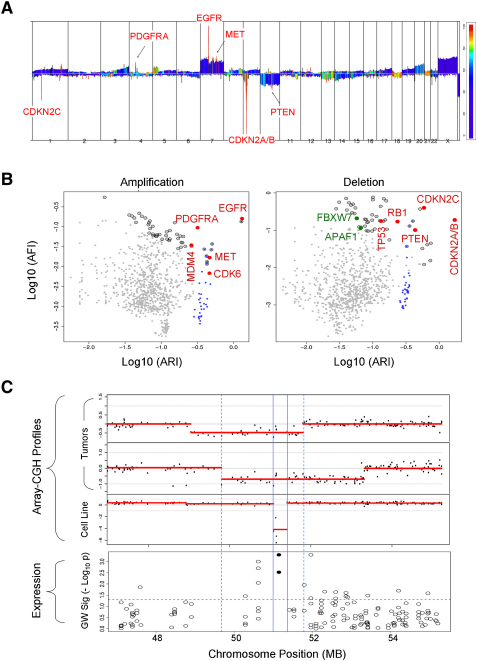
Analyses of GBM Oncogenome by GTS Identified *CDKN2C* Deletion (A) Skyline profile of GBM oncogenome with ARI on y axis. AFI is represented by color scale based on percentile rank, highlighting the most focally altered regions. (B) Two-dimensional GTS plots of log(ARI) (x axis) and log(AFI) (y axis) for amplification (left) and deletion (right). Black outer circles mark ROIs ranked among top 50 by GTS scores (Table 1). Red circles mark ROIs spanning signature events. ROIs on frequently gained chr7 are marked in blue on the amplification plot. Similarly, ROIs on chr10 are marked in blue on the deletion plot. Note separation of MET or PTEN from Chr7 or Chr10 events, respectively. (C) Deletion ROI spanning *CDKN2C* is present in 4/28 glioblastoma tumor samples (two examples shown), defining a focal minimal common region of deletion of 2Mbp (dashed lines). Homozygous deletions are common in glioma cell lines (5/18) and further refine the minimal common region of deletion to *p18^INK4C^* (solid lines). Analysis of copy-number-concordant expression by gene weight modeling (see Experimental Procedures) demonstrates highly significant coordinate loss of *p18^INK4C^* expression in tumors and cell lines that show chromosomal deletion. Gene weights for both probe sets for *p18^INK4C^* are significant at p < 0.01 (black circles). GW Sig, gene weight significance.

In two dimensions, array-CGH measures the relative changes in chromosomal copy number as a deviation from the baseline diploid state at every interrogated position along the genome. As such, it captures both the amplitude and width of a CNA in a tumor. Since the frequency of a CNA is believed to be a strong indicator of potential biological significance, recurrence is considered the third dimension for defining the topography of a CNA in GTS. We derived two indices to capture these three major features describing the topography of any given CNA. First, the Aberration amplitude and Recurrence Index (ARI) measures the composite contribution of the copy number value (log2 amplitude of the CNA) and the frequency of the aberration (recurrence) at a genomic position (See Experimental Procedures). Second, the Aberration Focality Index (AFI) weights the amplitude and recurrence inversely by an estimate of the number of genetic elements spanned by the CNA (see Experimental Procedures). Thus, the AFI assigns to each region in the genome a measure of the likelihood of any genetic element within the region representing the true target of genomic alterations across a sample set. With ARI and AFI calculated for each genomic position, we represented the GBM oncogenome in a typical skyline profile, with ARI on the vertical axis and AFI on the color-scale (Figure 1A). Not surprisingly, salient events in red (high AFI) with high amplitude and recurrence (high ARI) coincide with known common, high-amplitude and focal CNAs in GBM, including *EGFR*, *PDGFRA*, *MET*, *PTEN*, and *CDKN2A/B* (*p16^INK4A^/p15^INK4B^*).

Next, we generated two-dimensional GTS plots with each dot representing a region of interest (ROI) defined by peaks in the ARI and AFI (Figure 1B, see Experimental Procedures). Since high ARI and AFI represent focal events with high amplitude and recurrence, the well-known signature events in GBM were grouped in the upper right as expected (Figure 1B, red circles). The importance of focality in identifying candidate targets is highlighted by separation of ROIs spanning *MET* or *PTEN*, driven by their high AFI, from the background of regional but highly recurrent Chr7 gain or Chr10 loss, respectively (Figure 1B, clusters of blue dots correspond to Chr7 and Chr10 ROIs, respectively). Moreover, infrequent but focal CNAs could be identified on the GTS plots based on their high AFI score (y axis) despite low ARI (x axis), as were the cases of infrequent but focal deletion of *APAF1* and *FBXW7*, not previously described in GBM (Figure 1B, green circles).

To rank the likely significance of GTS-defined ROIs, we calculated a GTS score reflecting the combined contributions of ARI and AFI for each ROI and listed the top 50 rank-ordered amplifications and deletions, respectively, in Table 1. Additionally, since copy number aberration is a known mechanism to dysregulate expression, we generated RNA transcriptome profiles on a subset of samples to identify those ROI-resident genes whose expression patterns were concordant with their copy number. Here, we calculated a gene's gene weight (GW) (Aguirre et al., 2004) (see Experimental Procedures) and considered those with p values less than 0.05 as exhibiting copy-number-concordant expression (Table 1, in blue). By such GW criteria, 30% of ROI-resident genes (26/80 in amplified ROIs; 16/58 in deleted ROIs) showed copy-number-concordant expression, including all of the known signature events. Interestingly, among those not exhibiting such concordance are *FBXW7* and *APAF1*, pointing to additional common mechanisms for their inactivation in GBM (see Discussion).

### Codeletion of CDKN2C and CDKN2A via a Feedback Regulatory Circuit in Human GBM

The *CDKN2C* (*p18^INK4C^*) locus was identified by GTS as the top deletion peak in our data set (Table 1). Focused analysis of the array-CGH profiles (Figure 1C) revealed a clearly defined 436 kb minimal common region delimited by a homozygous deletion of the *p18^INK4C^* locus in a GBM cell line (solid lines), with slightly larger regions defined by CNAs detected in two primary GBM specimens (dashed lines). That *p18^INK4C^* is the target of these deletions was supported by finding that all 9 samples with this CNA showed reduction or loss of *p18^INK4C^* RNA expression (Figure S1), and gene weight modeling confirmed copy-number-concordant expression pattern for both *p18^INK4C^* probes (Figure 1C).

The observation of genomic deletion of *p18^INK4C^* was, at first glance, counterintuitive since all samples sustaining the *p18^INK4C^* deletion (n = 9 of 46; p = 0.009, Fisher's exact test) also harbored concurrent deletion of the *CDKN2A/CDKN2B* locus encompassing its related family members, *p16^INK4A^* and *p15^INK4B^*. Survey of a panel of 747 human cancer cell lines of 32 anatomical origins for genomic status of *CDKN2A* (*p16^INK4A^ and p14^ARF^*) and *CDKN2C* revealed that codeletion of these two loci was observed predominantly in glioma tumor cell lines (Table S3). Although protein expression by immunohistochemistry (IHC) does not inform the mechanism of nonexpression (e.g., pathological inactivation by genomic deletion versus normal physiological regulation), IHC analyses of p16^INK4A^ and p18^INK4C^ expressions on GBM tissue microarrays containing an independent set of GBM tumor specimens confirmed that a proportion of human GBM tumors (n = 10 of 59 informative cores) expressed low to undetectable levels of both proteins (Figure S2 and Table S4). Additionally, resequencing of *p18^INK4C^* in 53 human glioma cell lines (Table S5) identified three sequence variants of *p18^INK4C^* in *p16^INK4A^*-deleted cell lines. While we cannot definitively rule out the possibility that these represent rare germline variants without corresponding germline normal DNA, two of these three nonsynonymous sequence variants, p.F37I in GB-1 and p.A61D in KNS-60, targeted highly conserved or invariant amino acid residues (Figure S3).

Several lines of evidence suggest that the known principal RB pathway lesions in human tumors act in a mutual exclusive manner (Sherr and McCormick, 2002). However, in the mouse, it has been shown that loss of *p18^Ink4c^* and *p16^Ink4a^* can cooperate to induce pituitary tumor formation (Ramsey et al., 2007), suggesting that p18^Ink4c^ may have independent tumor suppressive activity in a pathway parallel to that of its related family member p16^Ink4a^. Along the same line, mice null for both *p15^Ink4b^* and *p16^Ink4a^/Arf* exhibited a broader tumor spectrum than mice deficient for *p16^Ink4a^/Arf* alone (Krimpenfort et al., 2007). On the other hand, the presence of an E2F binding site within the *p18^INK4C^* promoter (Blais et al., 2002) raised the possibility of a regulatory loop, where inactivation of *p16^INK4A^* in nascent cancer cells triggers a compensatory upregulation of *p18^INK4C^* via E2F, leading to genetic pressure for its concomitant or subsequent deletion.

**Figure 2:**
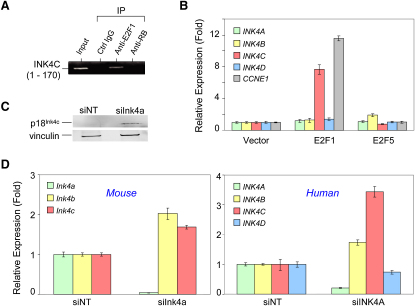
Acute Suppression of *p16^INK4A^* Results in Compensatory Upregulation of *p18^INK4C^* (A) E2F transcription factors bind the *p18^INK4C^* promoter in human astrocytes. A *p18^INK4C^* promoter DNA fragment was immunoprecipitated with anti-E2F1, but not with the control or an RB1 antibody. (B) Human astrocytes were transiently transfected with empty vector or expression constructs for E2F1 and E2F5. INK4 and *CCNE1* RNA expression was analyzed by RT-qPCR at 72 hr after transfection and normalized to *GAPDH* expression. Vector transfected cells were set to 1 for each transcript. E2F1, but not E2F5, specifically increased *p18^INK4C^* (INK4C) RNA to a similar extent as the well-characterized E2F target *CCNE1*. Error bars represent mean ± standard deviation. (C) Astrocytes from *p53^−/−^* mice at passage 2 were transfected with nontargeting siRNA (siNT) or siRNA targeting *p16^Ink4a^* (siInk4a) and assayed for p18^Ink4c^ protein expression at 72 hr after transfection. Loading control, vinculin. (D) Murine *p53^−/−^* astrocytes and normal primary human astrocytes were transfected with siNT or siInk4a and assayed for RNA expression levels of *p16^Ink4a^* (Ink4a), *p15^Ink4b^* (Ink4b), *p18^Ink4c^* (Ink4c), and *p19^Ink4d^* (Ink4d) by RT-qPCR at 72 hr posttransfection. Relative levels compared to Ink4 expression of siNT-transfected cells are shown after normalization to *Gapdh*. Error bars represent mean ± standard deviation.

To explore this hypothesis of a feedback circuit involving *p18^INK4C^* via E2F1, we performed chromatin immunoprecipitation and found that, in proliferating human astrocytes, the activating E2F1 transcription factor was indeed bound physically to the *p18^INK4C^* promoter (Figure 2A). Moreover, transient enforced expression of E2F1 in primary human astrocytes led to a specific increase in *p18^INK4C^* RNA, but not the other three related INK4 CKIs (Figure 2B). Acute suppression of *p16^Ink4a^* in immortalized (*p53*^−/−^) murine astrocytes resulted in a significant induction of p18^Ink4c^ protein (Figure 2C). Quantitative real-time RT-qPCR demonstrated that this is at least in part due to regulation of transcription or message stability because suppression of *p16^Ink4a^* by siRNA (siInk4a) resulted in a 2-fold increase of *p18^Ink4c^* RNA compared to control cells transfected with nontargeting siRNA (siNT) (Figure 2D), an effect that was maintained for up to 120 hr posttransfection (data not shown). Similarly, RT-qPCR analysis of normal human astrocytes showed a 3.5-fold increase in *p18^INK4C^* expression following *p16^INK4A^* knockdown (Figure 2D). A modest upregulation of *p15^INK4B^* was also observed while *p19^INK4D^* was not induced upon *p16^INK4A^* knockdown. Taken together, these data supported the view that a p16^INK4A^-E2F1-p18^INK4C^ feedback circuit is operative in the astrocytic lineage and underlies pressure for codeletion of these two related CKIs in GBM tumors.

### Functional Significance of p18^INK4C^ Inactivation in p16^INK4A^-Deleted GBM Cells

**Figure 3:**
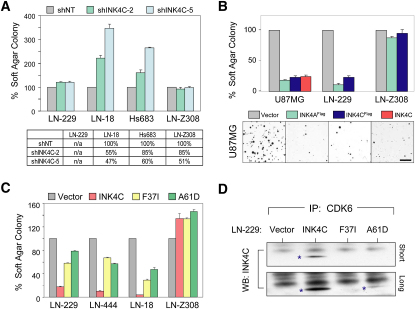
*p18^INK4C^* Inactivation in *p16^INK4A^* Null GBM Cell Lines Enhances Tumorigenicity (A) GBM cells, LN-229 (*p16^INK4A^/p18^INK4C^* null), LN-18, Hs683 (both *p16^INK4A^* null, *p18^INK4C^* WT), and LN-Z308 (*p16^INK4A^/p18^INK4C^* WT, *CDK4/Cyclin D1*-amplified) infected with retroviral expression constructs for small hairpins targeting *p18^INK4C^* (shINK4C-2 and shINK4C-5) or a nontargeting hairpin (shNT) were scored for colony formation in soft agar at day 14 and plotted as percentage of shNT controls. Error bars represent standard deviation of duplicates. Suppression of *p18^INK4C^* significantly increased the number of colonies in LN-18 (p = 0.01 and p = 0.006 for shINK4C-2 and shINK4C-5, respectively) and Hs683 (p = 0.07 and p = 0.004 for shINK4C-2 and shINK4C-5, respectively). Degrees of *p18^INK4C^* knockdown were determined by RT-qPCR relative to shNT-infected cells (100%) and indicated in the bottom table. Error bars represent mean ± standard deviation. (B) *p16^INK4A^/p18^INK4C^* null GBM lines U87MG and LN-229 as well as *p16^INK4A^/p18^INK4C^* WT, *CDK4/Cyclin D1*-amplified LN-Z308 stably infected with retroviral expression constructs for Flag-tagged p16^INK4A^ (INK4A^FLAG^), Flag-tagged p18^INK4C^ (INK4C^FLAG^), WT p18^INK4C^ (INK4C), or empty vector (Vector) were assayed for colony formation in soft agar. Soft agar colonies were stained and counted at day 14 and plotted as percentage ± standard deviation relative to vector-infected cells (U87MG-Vector 100%, U87MG-INK4C^FLAG^ 23 ± 2% [p = 0.0005], U87MG-INK4A^FLAG^ 18 ± 2% [p = 0.0003]; LN-229-Vector 100%, LN-229-INK4C^FLAG^ 23 ± 3% [p = 0.0002], LN-229-INK4A^FLAG^ 11 ± 2% [p=0.0001]). The scale bar indicates 2 mm. (C) GBM cell lines LN-229 (*p16^INK4A^/p18^INK4C^* null), LN-444, LN-18 (both *p16^INK4A^* null, *p18^INK4C^* WT), and LN-Z308 (*p16^INK4A^/p18^INK4C^* WT, *CDK4/Cyclin D1*-amplified) infected with vector control (Vector), WT p18^INK4C^ (INK4C), or p18^INK4C^ variants (F37I and A61D) were assayed for soft-agar colony formation, as in (B). Both p18^INK4C^ variants exhibited reduced capability of repressing colony formation. Error bars represent mean ± standard deviation. (D) Stable LN-229 cell populations were derived as in (C). Wild-type, but not mutant, p18^INK4C^ coimmunoprecipitated with CDK6 (p18^INK4C^ bands are marked by asterisks). The same effect was observed with binding to CDK4, albeit interaction between wild-type p18^INK4C^ and CDK4 was much weaker (data not shown).

To assess the functional significance of such a transcriptional feedback circuit, we asked whether p18^INK4C^ inactivation would further enhance the malignant properties of GBM beyond those conferred by *p16^INK4A^* loss (Table 2). Here, we made use of two established GBM cells, LN-18 and Hs683, which retain *p18^INK4C^* but lack *p16^INK4A^*. Using two shRNA constructs capable of knocking down *p18^INK4C^* RNA by 45%–53% (Figure 3A), *p18^INK4C^* depletion caused a 1.5- to 3.5-fold increase in anchorage-independent colonies in LN-18 and Hs683, respectively (Figure 3A). These *p18^INK4C^* shRNAs had no effect on soft-agar formation in *p18^INK4C^-*null LN-229 cells or in *Cyclin D1/CDK4*-amplified LN-Z308 cells, indicating that the phenotypes are not due to nonspecific shRNA effects. Similar results were obtained using synthetic *p18^INK4C^* siRNA oligos, where in the *p16^INK4A^* null and *p18^INK4C^*-WT cell lines LN-18 and LN-444, 50% knockdown of *p18^INK4C^* increased soft agar colony formation 2- and 8-fold, respectively (Figure S4). Again, *p18^INK4C^* knockdown did not increase colony formation in LN-Z308 cells (Table 2). Conversely, we examined the consequences of p16^INK4A^ and p18^INK4C^ reconstitution in U87MG and LN-229, two established GBM cells with concomitant deletions of the *p16^INK4A^* and *p18^INK4C^* loci as documented by copy number profiling and/or qPCR (data not shown). As expected, comparable levels of enforced expression of p16^INK4A^ and p18^INK4C^ in U87MG cells (Figure S5A) significantly inhibited proliferation (Figures S5B and S5C) and anchorage-independent growth in vitro (Figure 3B; Table 2). In contrast, enforced expression of p16^INK4A^ or p18^INK4C^ had no impact in LN-Z308 GBM cells with amplification and overexpression of *Cyclin D1* and *CDK4* and intact *p16^INK4A^* and *p18^INK4C^* genomic loci) (Table 2; Figure 3B; Figure S5C).

That the combined loss of *p16^INK4A^* and *p18^INK4C^* function confers enhanced malignant potential over that associated with *p16^INK4A^* loss alone in human GBM cells was further substantiated by demonstration that tumor-associated p18^INK4C^ variants occurring at highly conserved residues (p.F37I in GB-1 and p.A61D in KNS-60; Figure S3) were loss-of-function mutants. Specifically, reconstitution of these variants in *p18^INK4C^* null cell lines imparted at best 50% of suppressive activity of wild-type *p18^INK4C^* in both anchorage-independent growth and cell proliferation assays (Figure 3C; Figure S6; data not shown). Mechanistically, we found that the F37I and A61D variants did not bind CDK6 in coimmunoprecipitation studies (Figure 3D), thus providing a molecular basis for the loss-of-function phenotype of these mutant alleles.

### Inactivation of p18^Ink4c^ in p16^Ink4a^-Deficient Primary Astrocytes Conferred Tumorigenicity

**Figure 4:**
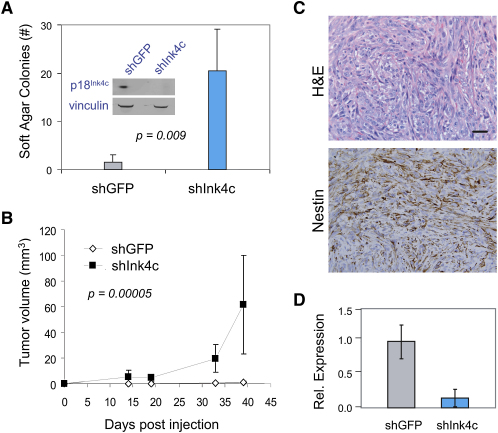
*p16^Ink4a^* and *p18^Ink4c^* Coinactivation Confers Tumorigenicity to Murine Astrocytes (A) Murine *p16^Ink4a^/ARF* null astrocytes were infected with lentiviral shRNA expression constructs targeting GFP (shGFP) or *p18^Ink4c^* (shInk4c). Knockdown of *p18^Ink4c^* resulted in drastically lower p18^Ink4c^ protein levels (loading control, vinculin) and significantly higher colony formation in soft agar (error bars indicate mean ± standard deviation of quadruples). (B) Stable astrocyte populations derived as in (A) were subcutaneously injected into Ncr nude mice. While *p18^Ink4c^*-deprived cells formed tumors within 2 weeks, control cells did not generate tumor (mean ± standard deviation plotted). Injection sites of control mice were histologically confirmed to be tumor free. (C) Tumors derived from shInk4c-infected astrocytes displayed malignant anaplastic histology and a high mitotic rate by H&E staining. Immunohistochemical analysis confirmed expression of the neural marker Nestin in a pattern similar to human astrocytomas. The scale bar indicates 30 μm. (D) Matrigel-embedded shGFP-infected astrocytes and tumor-derived shInk4c-infected astrocytes were assayed for *p18^Ink4c^* RNA expression relative to *Gapdh* expression confirming stable *p18^Ink4c^* knockdown in tumor cells. Error bars indicate mean ± standard deviation.

The molecular evidence of compensatory regulation between *p16^Ink4a^* and *p18^Ink4c^* in astrocytes suggested that complete deactivation of RB pathway tumor suppression activities in this cell type will require concomitant inactivation of both CKIs. To directly address this point, we compared in vivo tumorigenic potential of primary astrocytes that were inactivated for p16^Ink4a^/Arf alone or for p16^Ink4a^/Arf and p18^Ink4c^. Here, we used lentivirally delivered shRNA targeting *p18^Ink4c^* in primary nontransformed *p16^Ink4a^/Arf^−/−^* astrocytes to determine whether inactivation of *p18^Ink4c^* bestowed oncogenicity to these nontumorigenic primary cells. Reduction of *p18^Ink4c^* expression in already *p16^Ink4a^*-deficient primary astrocytes conferred anchorage-independent growth in vitro and turmorigenicity in vivo (Figure 4). Specifically, using two independent shRNA (data shown for one shRNA), near complete and stable knockdown of *p18^Ink4c^* (Figure 4A inset) in primary *p16^Ink4a^/Arf^−^*^/−^ astrocytes resulted in enhanced anchorage-independent growth in semisolid medium in culture. When transplanted subcutaneously into immunodeficient hosts, *p18^Ink4c^*-suppressed *p16^Ink4a^/Arf^−/−^* astrocytes formed malignant tumors that were strongly Nestin-positive, in contrast to controlled *p16^Ink4a^/Arf^−/−^* astrocytes which were not tumorigenic in vivo (Figures 4B and 4C). The resultant tumors maintained low to absent *p18^Ink4c^* expression at the RNA and protein levels (Figure 4D and data not shown). Enhanced in vivo tumorigenic phenotype upon *p18^Ink4c^* suppression was similarly observed in astrocytes deficient for *p16^Ink4a^/Arf* and *Pten* (Figure S7A). Furthermore, even in the presence of EGFRvIII oncogene, suppression of *p18^Ink4c^* trended toward development of larger tumors from *p16^Ink4a^/Arf^−/−^* astrocytes (Figure S7B). In conclusion, full transformation of primary astrocytes in a xenograft model required inactivation of p18^Ink4c^ in addition to that of p16^Ink4a^.

## Discussion

In this report, we analyzed the copy number and expression profiles of human GBM cell lines and tumors using a nonheuristic methodology called GTS. In addition to the well-known and highly recurrent events, GTS defines and ranks many previously unrecognized CNAs, uncovers frequent codeletion of two related CKI genes, *CDKN2C* and *CDKN2A*, in human cancers, and implicates *APAF1* and *FBXW7* in glioma pathogenesis.

Recent data in the mouse have challenged the dogma of exclusivity on involvement of major components of the CKI-CDK4/6-RB cell cycle regulatory axis in cancer. Ramsey et al. have provided evidence that loss of *p18^Ink4c^* can result in upregulation of *p16^Ink4a^* in specific murine tissues and that combined germline nullizygosity for both CKI facilitates the development of pituitary tumors (Ramsey et al., 2007). Correspondingly, Krimpenfort et al. have established compensatory p15^Ink4b^ protein stabilization in *p16^Ink4a^* null mice, supporting a cooperative tumor suppressor role for *p15^INK4B^* (Krimpenfort et al., 2007). Whether similar compensatory mechanisms among members of the Ink4 family of CKI held for humans was uncertain, particularly given the known cross-species differences in RB pathway regulation in the development of normal and neoplastic cells (Gil and Peters, 2006; Kim and Sharpless, 2006). Here, the collective genomic and functional evidence of this report provides the first documentation of common coinactivation of multiple members of CKIs in human GBM via genomic codeletion of the *CDKN2A/B* and *CDKN2C* loci or concurrent loss of protein expression of p18^INK4C^ and p16^INK4A^.

Importantly, the elucidation of underlying molecular circuitry driving above patterns of inactivation provides new insight into RB pathway function in human cancer. By demonstrating a p16^INK4A^-E2F-p18^INK4C^ feedback circuit operative in the astrocytic cell lineage, we provided molecular basis for “back-up” tumor suppressors such as p18^INK4C^. We showed that p18^INK4C^ backup tumor suppressor is engaged in the relatively common setting of p16^INK4A^ inactivation, resulting in enhanced proliferation and subsequent E2F-mediated induction of *p18^INK4C^* expression, consequently leading to genetic pressure for subsequent inactivation of p18^INK4C^. It is worth noting that, while its inactivation is necessitated, at least in part, by its redundant role with p16^INK4A^ in cell-cycle regulation, our results do not exclude the possibility that p18^INK4C^ may have additional functional activities beyond G1 CDK inhibition driving its inactivation during gliomagenesis. On the basis of these data, we conclude that p18^INK4C^ is a bona fide tumor suppressor in human GBM and that a hierarchy of tumor suppressive roles for members of the INK4 CKI exists, wherein p18^INK4C^ likely serves as a back-up to loss of p16^INK4A^. Such hierarchy of redundancy speaks further to the critical importance of intact RB pathway function in constraining human tumorigenesis.

GTS is a computational methodology for copy number data that incorporates focality with amplitude in context of frequency (recurrence) to determine likely significance of a given CNA. The two scores computed in GTS, ARI and AFI, describe key features of CNA across samples and provide highly complimentary information. ARI readily identifies genomic regions which are recurrently altered while disregarding the focality of CNA events: common gain of chromosome 7 and recurrent EGFR-region amplification are both identified as salient alterations in GBM. AFI scoring further distinguishes genomic regions that are altered focally versus regionally across samples, measuring the degree to which CNA is specifically targeting each point in the genome. Thus, the two scores comprising GTS summarize the continuum of CNA from wide regional alterations to the highly focal events, which may directly implicate candidate targets. In this study, AFI and ARI were given equal weight in delimiting ROIs, thus preferentially emphasizing high-amplitude focal aberrations over broader regional alterations; such preference reflects the fact that the former is more readily amenable to downstream workup, but not necessarily more important biologically.

The power of GTS lies in its ability to inform on focal but infrequent events that would otherwise be lost in methodologies that consider mainly amplitude and recurrence. Indeed, infrequent but focal CNAs can be highly informative as they may point to genes that are activated or silenced by other means, such as deletions of *APAF1* and *FBXW7*. *APAF1* maps to 12q22-23, a region associated with common LOH in GBM (Watanabe et al., 2003). APAF1 is a critical component of the apoptosome and caspase-9 activation (Li et al., 1997b). Inactivation of this pathway has been implicated in GBM as highlighted by recent work showing near universal upregulation of Bcl2L12, a regulator of apoptosis downstream of APAF1 (Stegh et al., 2007). *FBXW7* is a haplo-insufficient tumor suppressor gene (Mao et al., 2004) recently shown to be frequently inactivated in T cell lymphomas by mutation or deletion (Malyukova et al., 2007; Maser et al., 2007). As it encodes an F-box protein that is part of the ubiquitin protein ligase complex, FBXW7 has many known glioma-relevant client proteins including cyclin E, c-Myc, Aurora-A, Notch, and c-Jun. In addition to a large body of literature implicating the Myc network in GBM (Bredel et al., 2005), as well as presence of *MYC* amplification in GBM (Table 1, no. 6), *FBXW7* expression itself has recently been correlated with GBM patient survival (Hagedorn et al., 2007) although a direct pathogenetic role in GBM has not been established. Interestingly, *FBXW7* and *APAF1* do not exhibit copy number-driven expression profiles among GBM tumors, consistent with their infrequent occurrence, thus pointing to alternative more common mechanisms for their inactivation in GBM. Accordingly, *APAF1* has been known to be inactivated by methylation (Soengas et al., 2001) and *FBXW7* by inactivating point mutations (Moberg et al., 2001). These findings of infrequent but focal deletion coupled with non-copy-number-concordant expression pattern should motivate direct examination of the mechanism and roles of APAF1 and FBXW7 inactivation in GBM.

The GBM oncogenome is highly complex and harbors numerous CNAs, many of which presumably target yet-to-be-discovered GBM cancer genes. We have demonstrated here that GTS can address one critical need in the development of a functional map of GBM genetic targets: namely, to prioritize those genomic alterations that are likely to be of importance from among those that are more likely to be bystanders of the cancer process. In particular, GTS has prioritized 100 top-ranking ROIs, encompassing a total of only 138 resident genes representing a limited list of candidates for downstream functional validation. Among these 138 candidates are 42 that exhibited significant copy number-concordant expression patterns, including 10 validated GBM genes, pointing to high probability of biological relevance for the remaining 32 GW-significant candidates residing within these ROIs. On the other hand, as exemplified by *APAF1* and *FBXW7*, non-GW-significant residents may also represent targets of rare genomic events that are more commonly dysregulated by other mechanisms, rendering them similarly productive entry points for identification of mutations or epigenetic alterations. Downstream functional validation of these high probability candidates should yield novel GBM genes and potential targets for therapeutic intervention.

## Experimental Procedures

### Cell Lines and Tumors

Frozen tumor specimens (Table S1A) were obtained from the Memorial Sloan-Kettering Cancer Center tumor bank. All tumor specimens were collected after obtaining written informed consent preoperatively. This study was approved by the Institutional Review Board of the hospital. Each tumor was confirmed histopathologically to be grade IV glioblastoma. Glioma cell lines (Table S1B) were propagated in RPMI-1640 medium supplemented with 10% FBS and penicillin/streptomycin/amphotericin B. Normal human astrocytes were obtained from ScienCell and Cambrex and propagated in astrocyte medium (ScienCell). Murine astrocytes were propagated in DMEM supplemented with 10% FBS and penicillin/streptomycin/amphotericin B. DNA from tumors and cell lines was isolated with DNeasy (QIAGEN). RNA was isolated with Trizol (Invitrogen), digested with DNase (Promega), and purified with RNeasy (QIAGEN). siRNAs were purchased from Dharmacon and transfected using Lipofectamine 2000 (Invitrogen). Normal human brain RNA was purchased from Ambion.

### Proliferation and Tumorigenicity Assays

Soft agar assays were performed in duplicate or triplicate in 6-well plates. 5000 cells per well were seeded in regular medium containing 0.4% low-melting agarose on bottom agar containing 1% low-melting agarose in regular medium. After 14 days, colonies were stained with Iodonitrotetrazoliumchloride (Sigma) and counted. For bromodeoxyuridine (BrdU) labeling, cells were incubated with 10 μM BrdU (Sigma) in regular medium for 30 min. Cells were then ethanol-fixed, RNase-digested, and incubated with anti-BrdU (DAKO), followed by a FITC-labeled secondary antibody (DAKO) counterstained with propidium iodide and analyzed by flow cytometry. For in vivo tumorigenicity assays, 10^6^ genetically engineered astrocytes comixed with matrigel (Sigma) were transplanted subcutaneously into flanks of Ncr nude mice (Taconic) and followed for tumor development. Tumor size was measured by caliper by the same operator over time. At termination of the experiment, tumors were harvested and processed for pathological and molecular analyses. All animal experiments were approved by Harvard's Institutional Animal Care and Use Committee (IACUC). Statistical analysis was performed with a Student's t test.

### Expression Analysis

Protein (10–30 μg) was resolved on 4%–12% Bis-Tris gradient gels (Invitrogen), transferred to a PVDF membrane (Perkin Elmer), and incubated with antibodies against p18^INK4C^ (mouse monoclonal antibody, Cell Signaling; rabbit polyclonal antibody, LabVision), tubulin (Sigma), vinculin (Santa Cruz), and Flag epitope (Sigma). Quantitative PCR was performed on an Mx3000P cycler (Stratagene) using QuantiTect SYBR green (QIAGEN). Primers were obtained from SuperArray. Reactions were performed as triplicates or quadruples for both test and control primers. Relative expression was calculated with the ΔΔC_t_ method. Reverse transcription was performed with Superscript II (Invitrogen) and oligo-dT priming. Immunoprecipitation was carried out using standard techniques. One milligram of cell lysate was incubated with 2 μg of CDK6 antibody (Santa Cruz) overnight.

### Retroviral Constructs

*INK4A* and *INK4C* cDNAs were PCR-amplified from pCMV-p18 (Dr. Y. Xiong) and pFlag-p16 (Dr. C. Geisen) and cloned into pBabe-puro3 as both untagged and as Flag fusions. *INK4C* point mutations were introduced using QuikChange II (Stratagene). Three shRNA sequences were annealed (sequences available upon request) and cloned into pSuperRetroPuro (Oligoengine). Retrovirus was produced in Phoenix A cells, and target cells were infected at 48 hr and 72 hr past transfection in the presence of 5 μg/ml polybrene (Sigma). Infected cells were selected with 2.5 μg/ml puromycin for 4 days before being assayed. Lentiviral shRNA constructs targeting GFP and *p18^INK4C^* were obtained through the RNAi Consortium (TRC). Sequences are available from their website.

### Resequencing

GBM cell line DNAs were sequenced as previously described (Davies et al., 2005) by direct sequencing of each exon with intronic flanking sequences utilizing ABI big dye chemistry on ABI3730 machines. Each variant was confirmed in three independent sequencing assays. Primer sequences are available upon request.

### Array-CGH

Genomic DNA was processed, labeled, and hybridized onto Agilent's 60-mer array-CGH microarrays with 44K or 244K density (for performance comparison, see (Greshock et al., 2007) according to manufacturer's protocol. Processing of array-CGH data to generate a segmented profile by circular binary segmentation (CBS) (Lai et al., 2005; Venkatraman and Olshen, 2007) was as detailed elsewhere (Aguirre et al., 2004). Complete profiles are deposited on the GEO website under super-series accession no. GSE9200 (http://www.ncbi.nlm.nih.gov/geo/query/acc.cgi?acc=GSE9200).

### Genome Topography Scanning

GTS is performed in two stages, starting with array-CGH data which has been smoothed by CBS: (1) calculation of ARI and AFI for each aCGH probe position in the genome, followed by (2) identification of local peaks in the combined GTS scores (ARI^∗^AFI). Separate ARI and AFI are calculated for gain and loss. ARI is computed for each probe position as the mean of CBS-smoothed log2 ratios across all samples showing chromosomal gain, and then likewise for all samples showing loss. A focality-weighted ARI (*fw*ARI) is calculated for gain and loss similarly to ARI, but after weighting the smoothed log2 ratio at each probe position by the number of genetic elements (genes, microRNAs, etc.) spanned by the CNA event, accounting for linkage of CNA across multiple CBS segments (see Supplemental Experimental Procedures). AFI is the ratio of *fw*ARI/ARI. To identify focal CNA events, regions of interest (ROIs) are then defined by bounding local peaks in the combined GTS scores (ARI^∗^AFI, equivalent to fwARI, see Supplemental Experimental Procedures). In this analysis of GBM data set, log2 ratio was used directly in the calculation of ARI and equal weighting was applied to each genetic element (RefSeq gene list) for the AFI score. Transformation of copy number and differential weighting of genetic elements may be utilized depending on the application. GTS algorithm is available as an R package at http://cbio.mskcc.org/brennan and is in the process of being submitted to BioConductor.

### Expression Profiling and Gene Weight Significance Calculation

RNA expression profiling was performed at the Dana-Farber Microarray Core facility using the U133Plus2.0 chip (Affymetrix). Gene weight (GW) was calculated as previously described (Aguirre et al., 2004; Carrasco et al., 2006; Hyman et al., 2002). Briefly, for each gene probe set, GW of expression values for test set “T” compared to reference set “R” is calculated by:

$$GW_{Tvs.R}=\frac{\bar{T}-\bar{R}}{\sigma^{T}+\sigma^{R}}$$

where test and reference sets are defined by presence or absence, respectively, of CNA in the chromosomal region including the gene. Chromosomal amplifications are tested separately from deletions. Significance was determined by permuting sample labels for expression data (1000 permutations, p value ≤ 0.05). Genes with GW significance are considered exhibiting “copy number concordant expression.”

### Chromatin Immunoprecipitation

Chromatin immunoprecipitation (ChIP) was carried out using the EZ ChIP kit per manufacturer's protocol (Upstate). Briefly, genomic DNA from 5 × 10^6^ normal human astrocytes was formaldehyde crosslinked and sonicated in 300 μl of lysis buffer until the average DNA fragment length was 600 bp. One-hundred microliters of lysate, diluted 10-fold in ChIP dilution buffer, was used per IP reaction. Antibodies used were anti-E2F1 (Santa Cruz, sc-193), anti-RB (Santa Cruz, sc-50), and rabbit control IgG (NeoMarkers, NC-100-P). *INK4C* and *ACTNB* promoter fragments were amplified as described (Blais et al., 2002).

## Figures and Tables

**Table 1:**
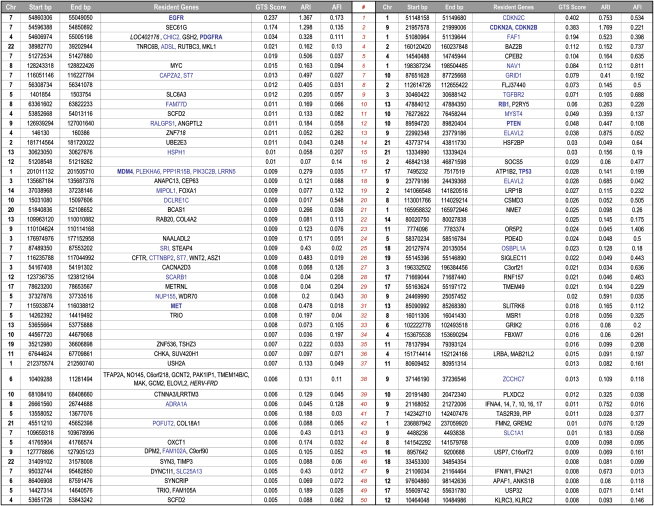
GTS Rank-Ordered ROIs

List of top 50 amplified (left) and deleted (right) ROIs, respectively, rank-ordered by GTS scores. ROI resident genes with gene-weight significance (p < 0.05) are highlighted in blue. Italics mark genes not represented on the expression array. Known GBM signature genes are in bold.

**Table 2:** Summary of p18^INK4C^ Gain-of-Function and Loss-of-Function Studies

				Tumorigenicity (Soft Agar Growth)	
	CDKN2A	p18^INK4C^	CCND1/CDK4	p18^INK4C^ Expression	p18^INK4C^ KD
LN-18	null	WT	WT	decrease	increase
LN-444	null	WT	WT	decrease	increase
Hs683	null	WT	WT	N/A	increase
LN-229	null	null	WT	decrease	no change
U87MG	null	null	WT	decrease	N/A
LN-Z308	WT	WT	amp	no change	no change

## Accession Numbers

Complete profiles are deposited on the GEO website under super series accession no. GSE9200 (http://www.ncbi.nlm.nih.gov/geo/query/acc.cgi?acc=GSE9200).

## Supplemental Data

Document S1. Supplemental Experimental Procedures and Seven Supplemental Figures

Document S2. Six Supplemental Tables Legends for Tables S1–S6 can be found in Document S1
