# Supplementary material for: Feedback Circuit among INK4 Tumor Suppressors Constrains Human Glioblastoma Development
Source: Cancer Cell. 2008 Apr 8;13(4):355–64. doi: 10.1016/j.ccr.2008.02.010 (PMC2292238; doi:10.1016/j.ccr.2008.02.010)
Supplement: Document S1. Supplemental Experimental Procedures and Seven Supplemental Figures [file mmc1.pdf]

Cancer Cell, Volume *13*

Supplemental Data

Feedback Circuit among INK4 Tumor Suppressors Constrains Human Glioblastoma Development

Ruprecht Wiedemeyer, Cameron Brennan, Timothy P. Heffernan, Yonghong Xiao, John Mahoney, Alexei Protopopov, Hongwu Zheng, Graham Bignell, Frank Furnari, Webster K. Cavenee, William C. Hahn, Koichi Ichimura, V. Peter Collins, Gerald C. Chu, Michael R. Stratton, Keith L. Ligon, P. Andrew Futreal, and Lynda Chin

Supplemental Experimental Procedures

Genome-Topography-Scanning:

***ACGH Profile Centering*:** Array-CGH log2 ratios carry information about relative copy number between regions but cannot determine absolute copy number. Therefore the data must be centered to a level which is considered "functionally euploid". After CBS segmentation, "segment-smoothed" profiles are generated by replacing the raw log2 ratio at each probe location with the mean of all neighboring probes in the same segment (S^{mean}). The profiles are then individually centered by the mode of the distribution of segment-smoothed log2 ratios.

Combining 44K and 244K aCGH data: 98% of the probes on the Agilent 44K array are also present on the 244K array. CBS-smoothed data acquired from the 44K array was resampled at the remaining genomic probe positions of the 244K array.

Gain/Loss thresholds: After centering, the distribution of all combined segment-smoothed profiles shows a sharp central peak around zero. Gain and loss thresholds are set at +/-0.2, approximately 10 SD from the middle 50%ile of the data centered at zero.

ARI score: Scores for gain and loss are calculated separately for each probe position as the absolute value of the sum of the segment-smoothed log2 ratios of all samples where the values are >0.2 or <-0.2, respectively.

AFI score: Aberration Focality Index measures what proportion of the ARI score is distributed per potential target genes or other genetic elements spanned by the region. AFI is the ratio of a focality-weighted ARI to unweighted ARI (*fw*ARI/ARI). As with ARI, AFI is calculated for each genomic position, separately for gain and loss samples. Focality weighting is performed with a conceptual model for the biological process of amplification and deletion that incorporates two fundamental aspects: (1) that CNA can progress in stage-wise fashion with progressive accumulation of extra copies associated with narrowing of the altered region, and (2) that DNA rearrangement within and across chromosomes may join nonadjacent sequence or delete intervening sequence such that a single amplicon may include non-contiguous genomic regions and be falsely represented as distinct CNAs in the aCGH profile. We consider three models for potential linkage of CNA across the profile: local, chromosomal and genomic. Local linkage treats each group of adjacent gained (or lost) segments as a contiguous discrete amplicon (or deletion) implying that the target genetic elements are spanned by the group of adjacent segments; Chromosome linkage considers that non-adjacent CNAs within the same chromosome represent a single amplicon (or deletion) with a shared set of targets. Genome linkage treats all CNA as if it belongs to a single complex amplicon (or deletion). Genome linkage is a conservative model, though not likely to be biologically accurate in most cases. Chromosomal linkage models the formation of amplicons with internal deletion, such as the co-amplification of CDK4 and MDM2 which typically excludes the adjoining region. Chromosomal linkage was used for the analysis in this study. Calculation of AFI is as follows:

For each segment $S_{i=1..N_{total}}$ in the profile of N_{total} segments:

S_i^{mean} = mean $\log_2$ ratio for segment *I*

$S_i^{elements}$ = number of genomic elements spanned by segment genomic start/end (or 1 if no elements are spanned)

Groups of potentially linked segments, S_{G_1}, S_{G_2}, etc., are determined by the linkage model:

Genome linkage: one group, S_G, comprised of all gained (or lost) segments

Chromosome linkage: 24 groups, $S_{G_{1..24}}$, of all gained (or lost) segments per chromosome

Local linkage: M groups, $S_{G_{1..M}}$, of contiguous gained (or lost) segments bounded by non-gained segments or chromosomal ends

Then for each group of segments, S_{G_n} , the N segments are ordered $(1 < i < N)$ by increasing $\left|S_i^{mean}\right|$. The segment focality-weighted mean, *fwMean*, is then calculated for each segment in the group by:

$$S_i^{fwMean} = \frac{\left(S_i^{mean} - S_{i-1}^{mean}\right)}{\sum_{j=i}^{N} S_j^{elements}}$$

After all profiles have been analyzed, focality-weight ARI (*fw*ARI) is calculated as for ARI, but using the S^{fwMean} of each segment instead of mean $\log_2$ ratio, S^{mean} .

Then AFI=*fw*ARI/ARI.

Peak selection and ROI bounding. The dual indices ARI and AFI are determined for each point in the genome and can be used directly to select genomic regions enriched for gene targets of CNA. For the purpose of summarizing the distribution of these target-enriched regions, a heuristic algorithm was developed to select regions of interest (ROIs) bounding peaks in the product of the two indices: ARI x AFI, which is equivalent to fwARI. Local peaks in fwARI are analyzed and ROIs are bounded at falloff of 75% peak maximum, or at the minimum to the next peak, whichever is narrower. Each ROI is annotated by the mean ARI and AFI indices for the region, and sorted by the product of mean indices. ROIs are flagged if over half of the probes in the ROI lie within a region previously reported to be a copy number variation (CNV) in one of the 40 studies compiled for build hg17 in version 1 of the Database of Genomic Variants (http://projects.tcag.ca/variation/) (Iafrate et al., 2004).

IHC on tissue microarrays.

Tissue microarrays (Cybrdi) containing 60 cores from 30 GBM tumors and 3 cores from normal brain were subjected to immunohistochemical analyses of $p16^{INK4A}$ (anti-$p16^{INK4A}$ antibody: Cell Signaling) and $p18^{INK4C}$ (anti-$p18^{INK4C}$ antibody: Cell Signaling) protein levels. Each core was examined and assigned a 0 to 4 score by an expert neuropathologist (K.L.L.) based on following parameters:

0 = 0% cells stain positive

1 = <10% of cells stain positive (scattered positive cells)

2 = 10-50% of cells stain positive

3 = 50-80% of cells stain positive

4 = >80% of cells stain positive

In addition, intensity of staining was scored as 0 to 3 for absent, weak, moderate and strong (Supplemental Table S4). Since normal brain cores were scored 0 or 1 for both proteins, cores with scores of 0 or 1 were assigned a "negative" call for expression. Staining intensities of all negative cores were considered weak or absent. While duplicate cores from the same tumor generally received the same call (positive or negative), in the case of $p18^{INK4C}$, cores from 4 cases were assigned different calls. Since basis for such this discordance cannot be determined and may be due to technical or biological variation (such as intratumoral heterogeneity), each core was considered an independent sample for this analysis. In parallel, these TMA sections were scanned by iCys laser scanning cytometer and analyzed to provide quantitative percentage of cells in each core that exhibited immunoreactivity.

Competitive PCR analysis of the genomic status of $p16^{INK4A}$ and $p18^{INK4C}$

Competitive PCR was carried out for all exons of $p16^{INK4A}$ and $p18^{INK4C}$ to identify homozygous deletions. Between four and eight primer pairs were designed to each exon before being tested against a series of twenty control primer pairs. For each exon the combination that gave the best multiplex PCR result with products of equal intensity was then used to screen the core cell line set. Single exon deletions of *ARF* exon 1 and $p16^{INK4A}$ exon 1 were confirmed using a different primer set in a non-multiplex assay. PCR conditions are listed in Methods and primer sequences are listed in Supplemental Table S6.

Figure S1. (A) Standardized gene expression versus regional log2 copy number for $p16^{INK4A}$ and $p18^{INK4C}$ in tumors (circles) and cell lines (triangles). $p16^{INK4A}$ shows frequent chromosomal deletion with corresponding low-level expression. $p18^{INK4C}$ also demonstrates this pattern in samples showing chromosomal loss or deletion (red). Standardized expression is calculated by the mean of all gene-specific probesets (U133Plus2.0, Affymetrix), mean-centered and scaled by standard deviation across all samples. Log2 ratio copy number is shown for segmented aCGH data (see Methods). Samples with segment log2 ratio < -0.2 (vertical dashed line) are considered to have chromosomal loss, and segment log2 ratios < -0.8 are characteristic of homozygous deletion. (B) $p16^{INK4A}$ and $p18^{INK4C}$ RNA expression in our dataset of GBM tumors based on Affymetrix U133Plus2.0 expression profiling. Each dot represents an individual tumor or normal human brain control. Axes depict absolute expression values derived from normalized array data. Note low $p16^{INK4A}$ and $p18^{INK4C}$ expression of normal human brain. (C) $p16^{INK4A}$ and $p18^{INK4C}$ RNA expression in our dataset of glioma cell lines based on Affymetrix U133Plus2.0 expression profiling. Each bar represents an individual glioma cell line or astrocyte control (NHA). X-axis depicts absolute expression values derived from normalized array data. $p18^{INK4C}$-null cell lines are circled.

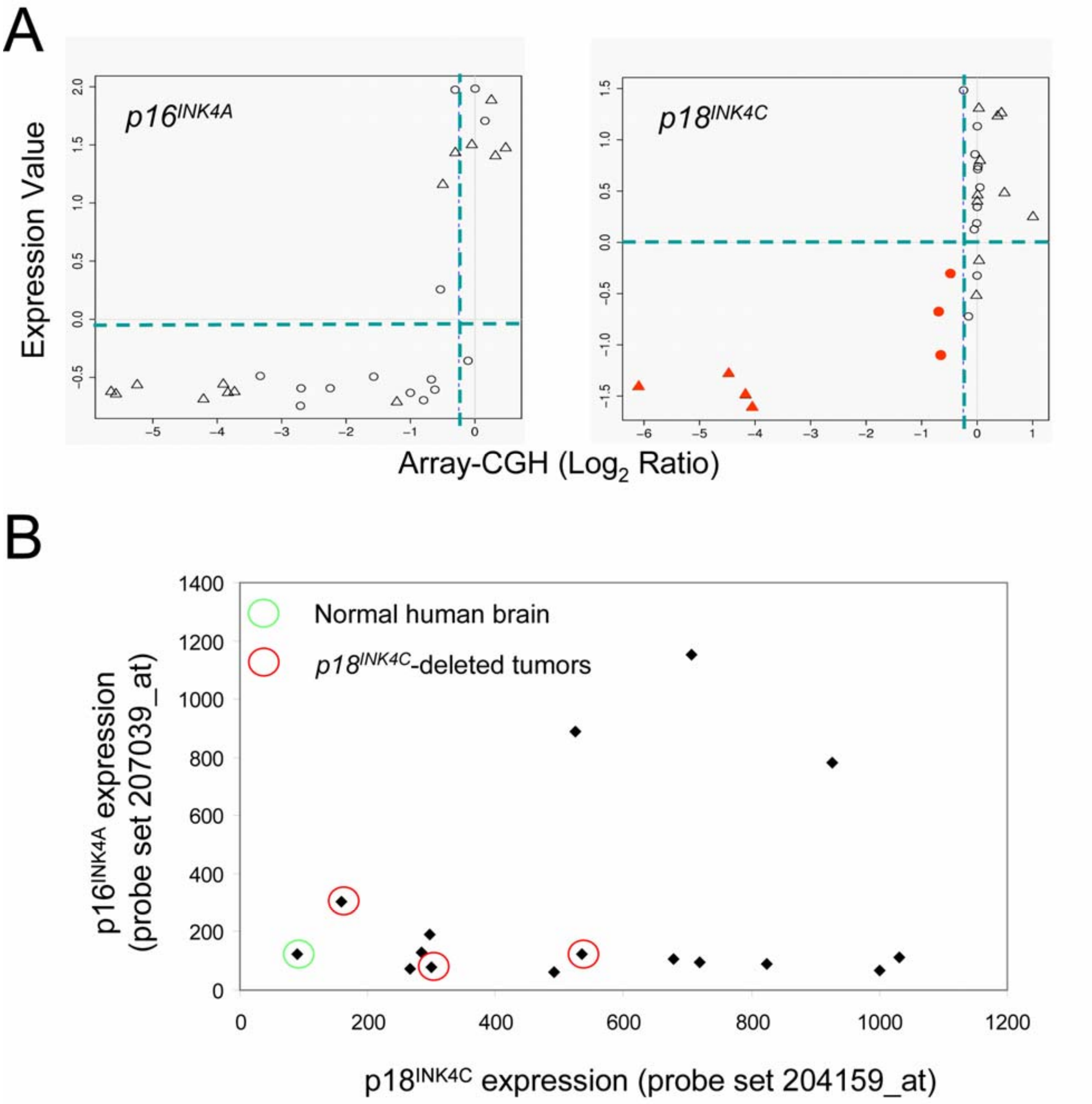

Supplemental Figure S1

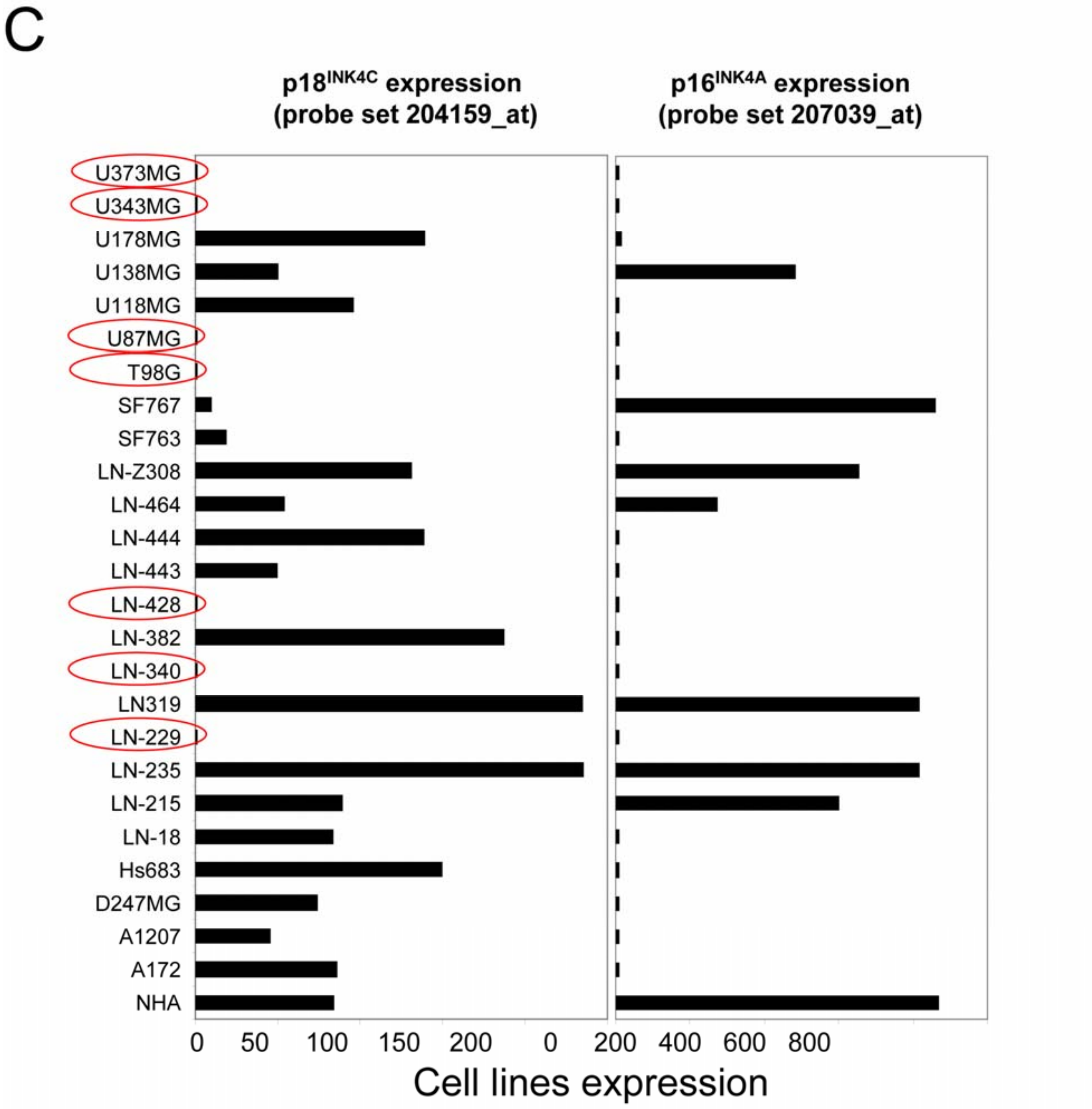

Supplemental Figure S1

Figure S2. Immunohistochemistry against p16^{INK4A} and p18^{INK4C} was performed on tissue microarrays containing 60 tissue cores of clinically annotated GBM tumor materials. Tumor cores were assigned a positive (Pos) or negative (Neg) call for expression of p18^{INK4C} and p16^{INK4A} (Supplemental Table S4). 25% of the informative cores were negative for p18^{INK4C} protein expression and 67% of these also were negative for p16^{INK4A} protein expression. Representative photomicrographs are shown (the scale bar indicates 30μm), on left, normal brain (core 61): negative for both p16^{INK4A} and p18^{INK4C}; in the middle: GBM (core 52): negative for both p16^{INK4A} and p18^{INK4C}; and on right: GBM (core 7): positive for both p16^{INK4A} and p18^{INK4C}.

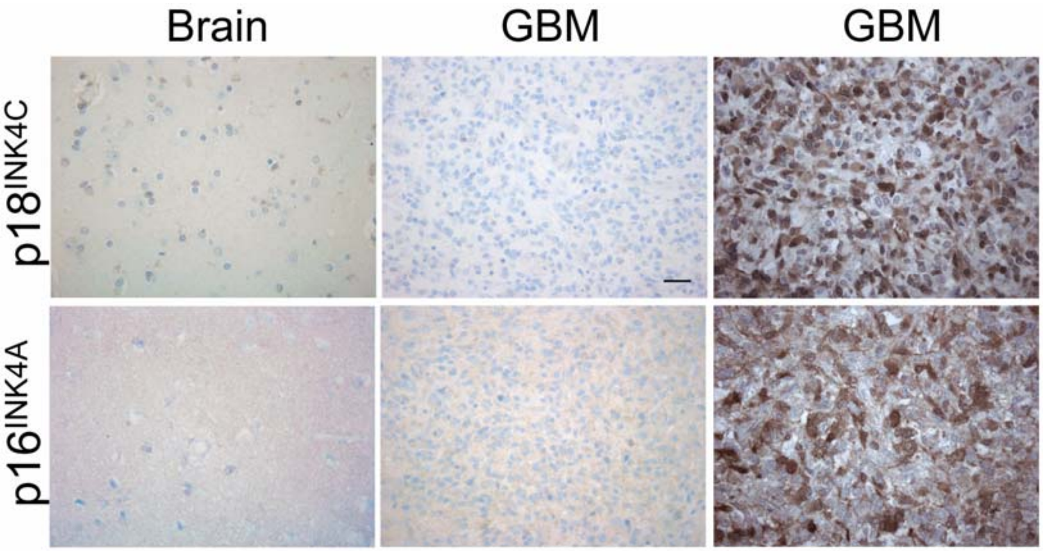

P18 Expression	Neg	Pos	Total
# Cores by Manual Scoring	15	44	59
Median % Staining by iCys	2%	42%	

P16 Expression	Neg	Pos
# Cores by Manual Scoring	10	5
Median % Staining by iCys	0%	62%

Supplemental Figure S2

Figure S3. *p18*^{INK4C} sequence variants in GBM. Sequencing of the *CDKN2C* (*p18*^{INK4C}*)* gene in the human GBM cell lines GB-1 and KNS-60 yielded two heterozygous sequence variants resulting in amino acid changes. GB-1 has an A-T nucleotide change in codon 109, resulting in a phenylalanine to isoleucine change (p.F37I). KNS-60 harbors a C-A nucleotide substitution in codon 182, altering the alanine residue at position 61 of the protein to aspartic acid (p.A61D). Both residues are highly conserved among INK4 members as shown by amino acid sequence alignment. A further less well conserved variant at glutamine 157c.470A>C p.Q157P was found in the KALS-1 glioblastoma line (data not shown).

GB-1 c.109T>A p.F37I

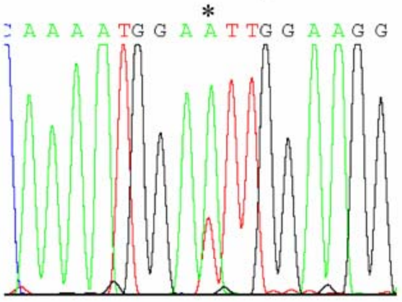

KNS-60 c.182C>A p.A61D

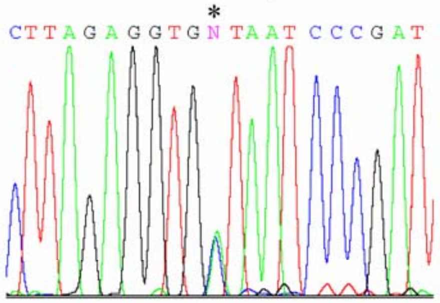

Species/Gene	I / D	AA	Accession
Human CDKN2C	VNVNAQNGFGRTALQVMKLGNPEIARRLLLRGANPDLKD	67	gi_17981699
Macaque CDKN2C	VNVNAQNGFGRTALQVMKLGNPEIARRLLLRGANPDLKD	67	gi_109004755
Mouse Cdkn2c	VNVNAQNGFGRTALQVMKLGNPEIARRLLLRGANPNLKD	67	gi_6680910
Human CDKN2A	ALPNAPNSYGRRPIQVMMMGSARVAELLLLHGAEPNCAD	74	gi_4502749
Mouse Cdkn2a	VSPNAPNSFGRTPIQVMMMGNVHVAALLLNYGADSNCED	66	gi_98986447
Opossum CDKN2A	TNPNAVNRFGRSAIQVMMMGNVRLAAILLQYGAEPNTPD	108	gi_77539754
Chicken CDKN2A	ADPNAVNSFGRTPIQVMMLGSPRVAELLLQRGADPNRPD	71	gi_45383299
Human CDKN2B	ADPNGVNRFGRRAIQVMMMGSARVAELLLLHGAEPNCAD	76	gi_17981694
Mouse Cdkn2b	ADPNALNRFGRRPIQVMMMGSAQVAELLLLHGAEPNCAD	68	gi_6671728
Pig CDKN2B	ADPNGLNHFGRRPIQVMMMGSARVAELLLLHGADPNCAD	69	gi_55742780
Cow CDKN2B	VDPNRLNRFGRRPIQVMMMGSARVAELLLLHGADPNCAD	69	gi_115497500
Xenopus CDKN2B	IPVNATNSHGRTPIQVMMMGSPKMAQLLLDHGADPKLPD	66	gi_55741980
Human CDKN2D	VHPDALNRFGKTALQVMMFGSTAIALELLKQGASPNVQD	71	gi_4502753
Macaque CDKN2D	VHPDALNRFGKTALQVMMFGSTAIALELLKQGASPNVQD	71	gi_109123370
Mouse Cdkn2d	VHPDALNRFGKTALQVMMFGSPAVALELLKQGASPNVQD	71	gi_31981844
Cow CDKN2D	VHPDVLNRFGKTALQVMMFGSPTIALELLKQGASPNVQD	71	gi_114051377

Supplemental Figure S3

Figure S4. GBM cell lines LN-18, LN-444 (both $p16^{INK4A}$-null, $p18^{INK4C}$-wt) and LN-Z308 ($p16^{INK4A}/p18^{INK4C}$-wt, *CDK4/Cyclin D1*-amplified) were transfected with non-targeting siRNA (siNT) or siRNA targeting $p18^{INK4C}$ (siINK4C). Cells were seeded into soft agar 48h after transfection. Colonies were stained and scored after 14d. The scale bar indicates 1mm. Error bars represent mean ± standard deviation of triplicates. RNA from transfected cells was harvested 72h after transfection and $p18^{INK4C}$ expression levels normalized to *GAPDH* were determined by RT-qPCR. Expression levels of siNT-transfected cells were set to 100% (error bars represent mean ± standard deviation).

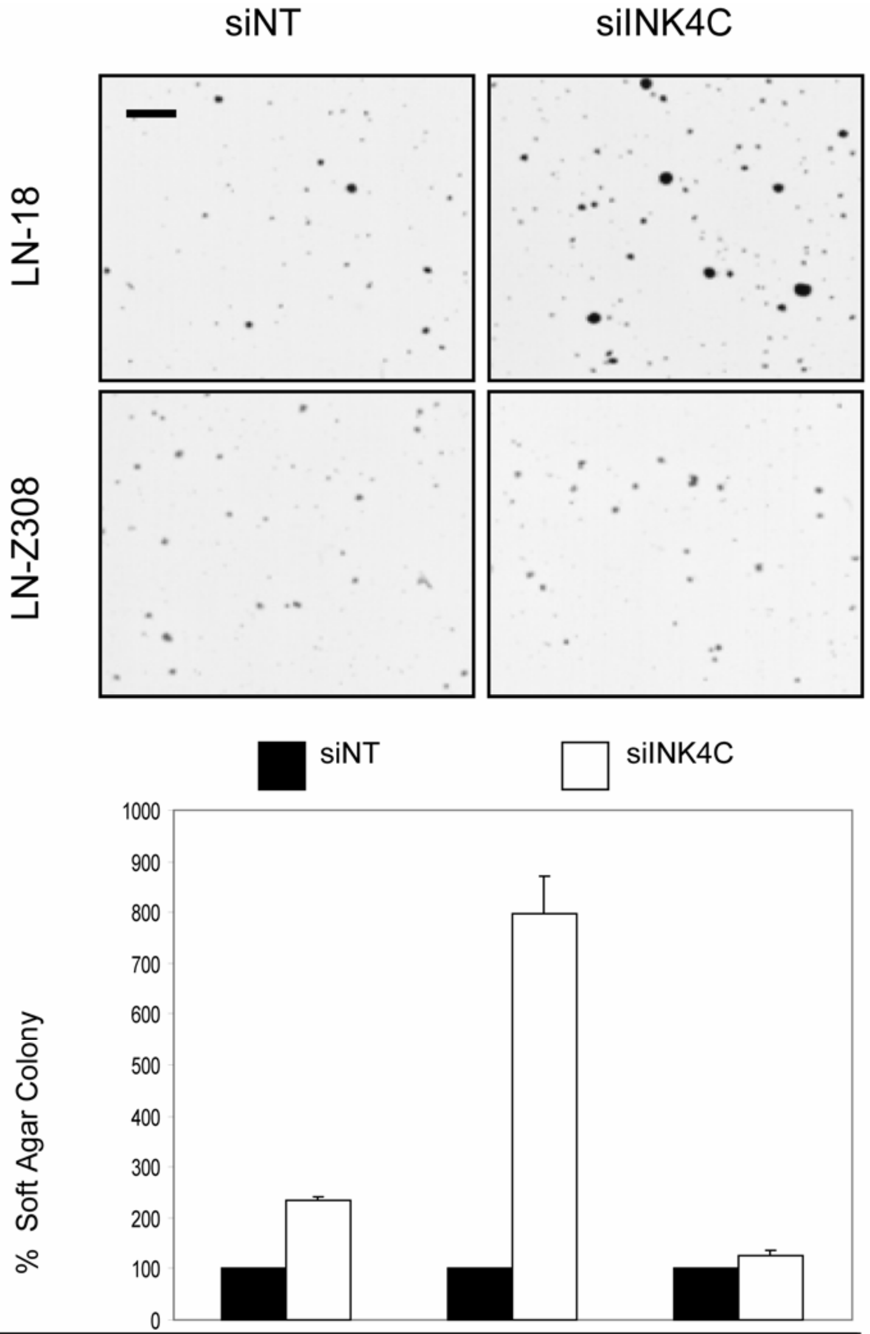

	LN-18	LN-444	LN-Z308
siNT	100%	100%	100%
siINK4C	48%	52%	38%

Supplemental Figure S4

Figure S5. (A) U87MG cells stably infected with retroviral expression constructs encoding Flag-tagged $p16^{INK4A}$ (U87MG-$INK4A^{FLAG}$), Flag-tagged $p18^{INK4C}$ (U87MG-$INK4C^{FLAG}$) or empty vector (Vector) were documented to express $p16^{INK4A}$ and $p18^{INK4C}$ at comparable level on Western blotting. Both U87MG-$INK4A^{FLAG}$ and U87-$INK4C^{FLAG}$ cells exhibited an enlarged, spread-out morphology whereas U87MG-Vector cells resembled the parental cell line U87MG, which forms spheroid three-dimensional structures under normal growth conditions *in vitro*. The scale bar indicates 60μm.

(B) Reduced proliferation of U87MG cells reconstituted with either $p16^{INK4A}$ or $p18^{INK4C}$. Stable, polyclonal cell populations were derived as described in (A) and assayed for proliferation under normal growth conditions *in vitro*. Mean values ± standard deviation are plotted.

(C) The $p16^{INK4A}/p18^{INK4C}$-null GBM line LN-229 as well as $p16^{INK4A}/p18^{INK4C}$-wt *CDK4*-amplified LN-Z308 cells stably infected with retroviral expression constructs for Flag-tagged $p16^{INK4A}$ ($INK4A^{FLAG}$), Flag-tagged $p18^{INK4C}$ ($INK4C^{FLAG}$) or empty vector (Vector) were BrdU-labeled and subjected to flow cytometry [X-axis: PI, Y-axis: FITC (BrdU)]. % of BrdU-positive cells is indicated (average of duplicates) for LN-229 and LN-Z308. $p16^{INK4A}$ or $p18^{INK4C}$ reconstitution effected dramatic reductions in S phase fractions from 44% to 2% or 10%, respectively, in LN-229 ($p<0.0001$).

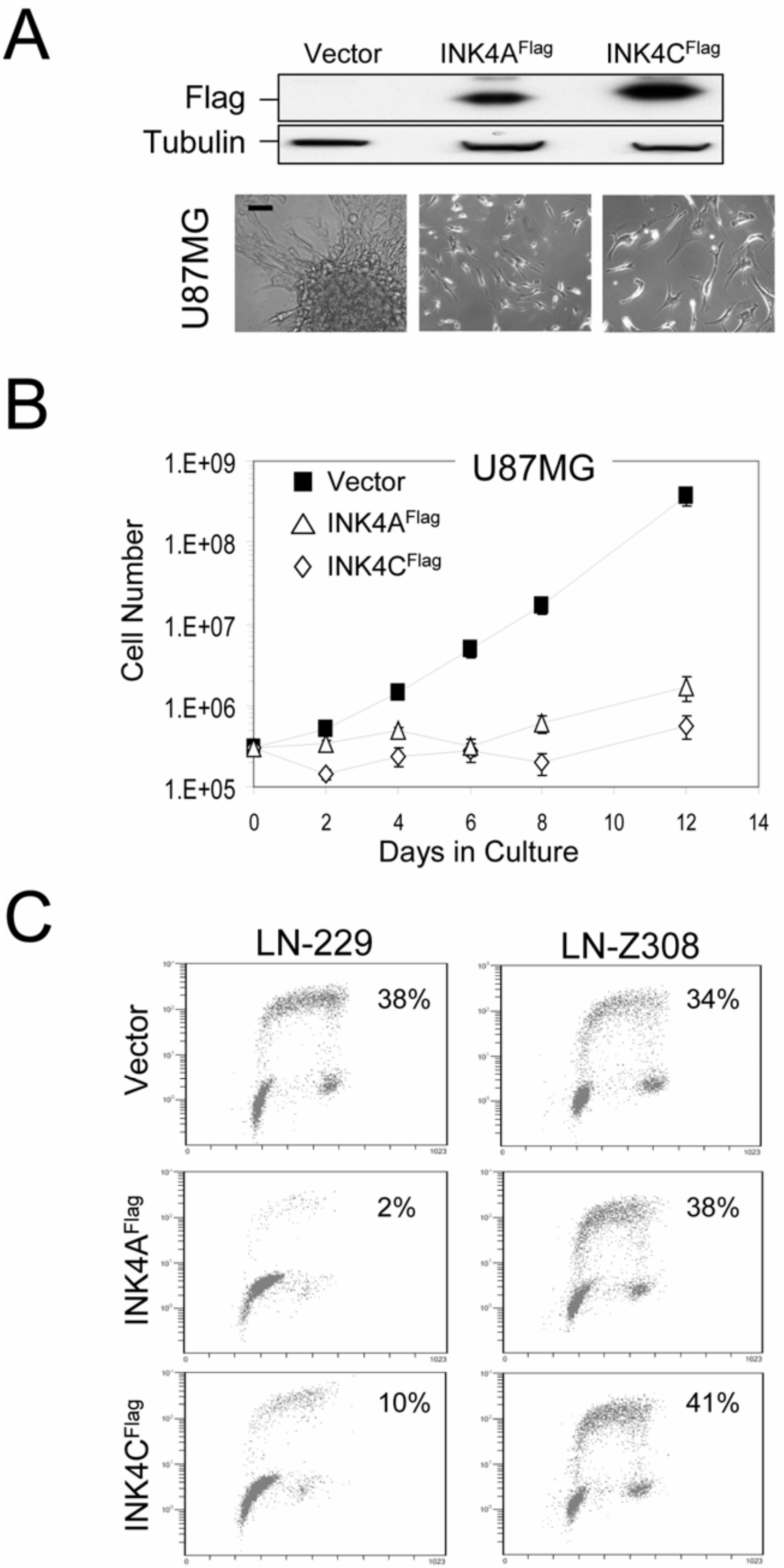

Supplemental Figure S5

Figure S6. p18^{INK4C} loss-of-function mutants. GBM cell lines LN-229 (*p16^{INK4A}/p18^{INK4C}*-null), LN-444, LN-18 (both *p16^{INK4A}*-null, *p18^{INK4C}*-wt) and LN-Z308 (*p16^{INK4A}/p18^{INK4C}*-wt, *CDK4/Cyclin D1*-amplified) were infected with empty retroviral vector (Vector) or expression constructs for wt p18^{INK4C} (INK4C^{WT}) or mutant p18^{INK4C} (INK4C^{F37I} and INK4C^{A61D}). Stable polyclonal cell populations were seeded into soft agar and colonies were stained and photographed after 14d. The scale bar indicates 2mm.

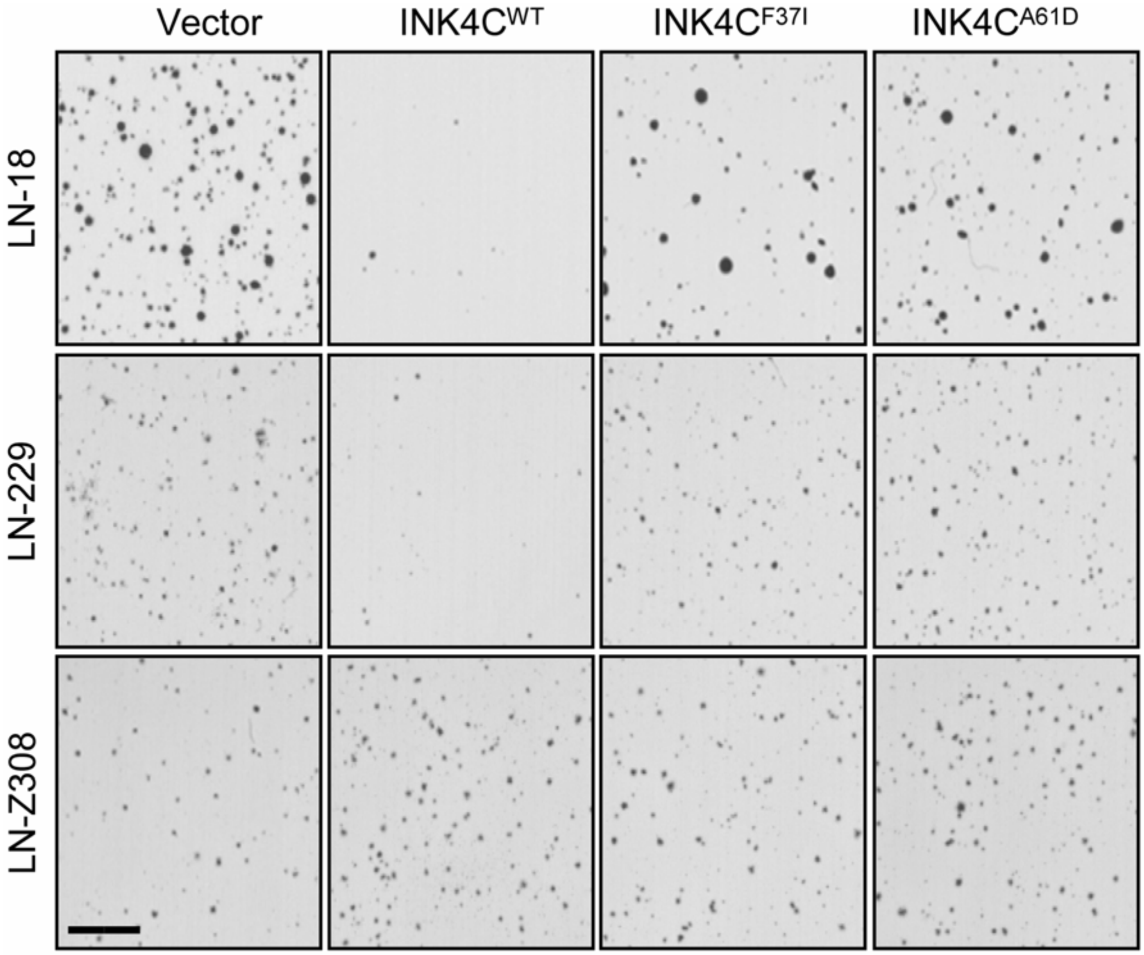

Supplemental Figure S6

Figure S7. (A) Murine *p16*^{Ink4a}*/Arf/Pten*-null astrocytes were infected with lentiviral expression constructs targeting GFP (shGFP) or *p18*^{Ink4c} (p18Lenti-sh1, p18Lenti-sh2). Stable astrocyte populations were subcutaneously injected into Ncr nude mice. Tumor burden per mouse at day 40 post injection is documented. Error bars represent mean ± standard deviation of at least 8 tumors. Both hairpins targeting p18^{Ink4c} dramatically increased tumor incidence and growth (p18Lenti-sh1: p = 0.0065, p18Lenti-sh2: p = 0.09). Penetrance: shGFP: 2/8, p18Lenti-sh1: 10/10, p18Lenti-sh2: 10/10. (B) Murine *p16*^{Ink4a}*/Arf*-null astrocytes expressing the EGFRvIII mutant were infected with lentiviral shRNA constructs targeting GFP (shGFP) or *p18*^{Ink4c} (p18Lenti-sh1, p18Lenti-sh2). Stable astrocyte populations were subcutaneously injected into Ncr nude mice. Tumor burden per mouse at day 33 post injection is documented. Error bars represent mean ± standard deviation of at least 8 tumors. Knockdown of *p18*^{Ink4c} resulted in larger tumors (p18Lenti-sh1: p=0.08, p18Lenti-sh2: p=0.01), and expression of *p18*^{Ink4c} (determined by RT-qPCR relative to *GAPDH*, error bars represent mean ± standard deviation) remained low in resultant tumors, confirming selection for inactivation of *p18*^{Ink4c} during tumorigenesis.

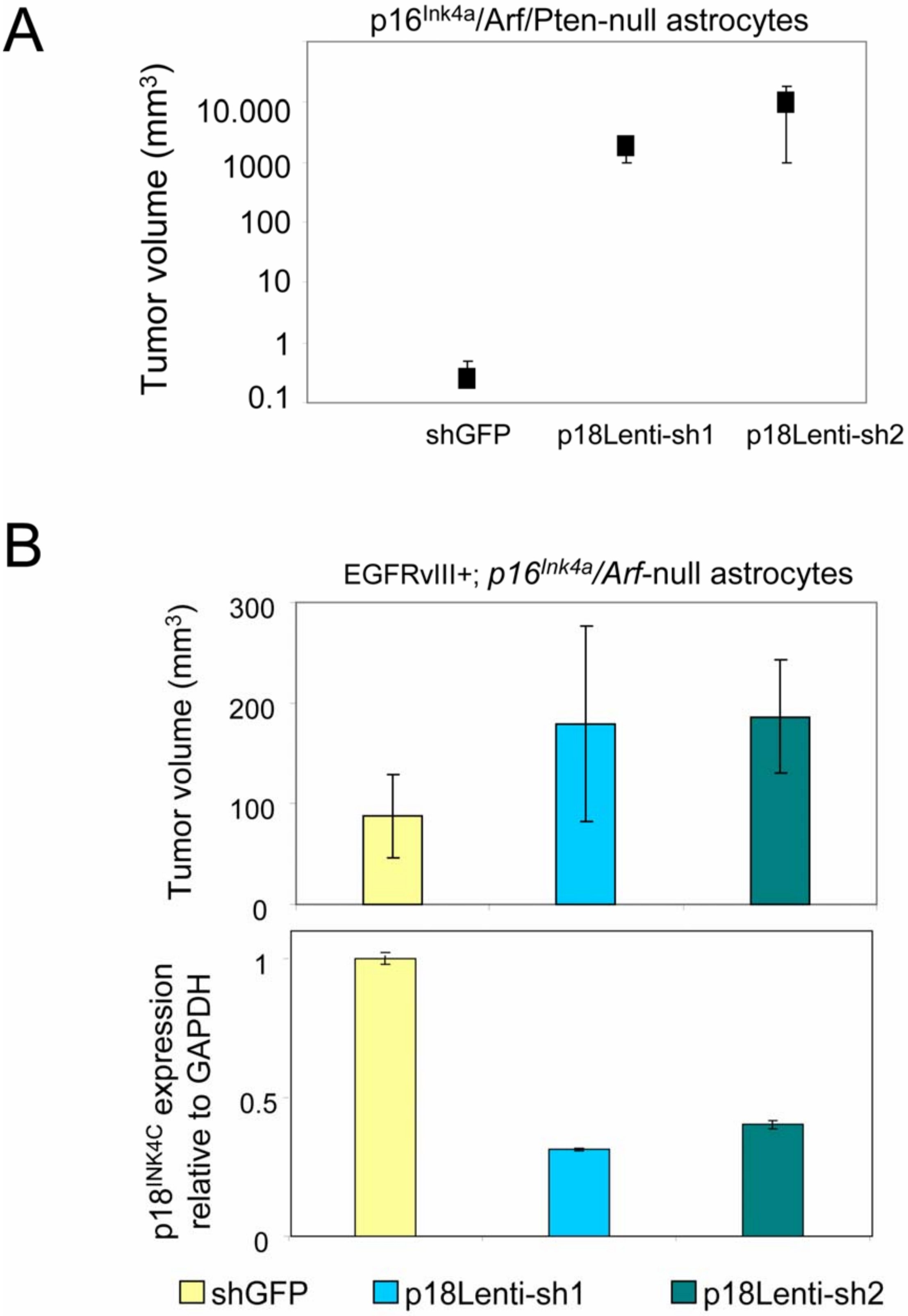

Supplemental Figure S7

Table S1: Signature mutations in our dataset of GBM tumors and cell lines (Furnari et al., 1997; Ishii et al., 1999).

Table S2: List of 42 focal and recurrent MCRs and their cytogenetic localization (column 2), nucleotide position (columns 3-4, based on human genome build 35), physical size of the MCR (column 5, in base pairs, bp), peak amplitude (column 6, in log2 units), recurrence among 36 CGH samples (column 7) and 18 tumors (column 8), number of resident genes in the MCR (column 9), number of genes represented on the Affymetrix expression array (column 10), number of genes showing copy number-driven expression (column 11), known cancer genes in the MCR (column 12, GBM signature genes in bold) and published copy number polymorphisms (CNP) in that region (column 13 (Conrad et al., 2006; Hinds et al., 2006; Iafrate et al., 2004; McCarroll et al., 2006; Sebat et al., 2004; Sharp et al., 2005; Tuzun et al., 2005)).

Table S3: Genomic status of $p16^{INK4A}$, *ARF* and $p18^{INK4C}$ in a panel of 747 human cancer cell lines of 32 anatomical origins (indicated in column labeled "Tissue") determined by competitive PCR. "# Lines" indicates the total number of cell lines of that Tissue origin analyzed, and the numbers in the columns labeled "ARF", "$\mathrm{p16}^{\mathrm{INK4A}}$" and "$\mathrm{p18}^{\mathrm{INK4C}}$" refer to the number of cell lines with homozygous deletions of that corresponding gene (in the case of $\mathrm{p16}^{\mathrm{INK4A}}$ truncating mutations detected by sequencing are included). The column "Remarks" states whether deletions of $p16^{INK4A}$ and $p18^{INK4C}$ occur in the same cell lines.

Table S4: Results of $\mathrm{p16}^{\mathrm{INK4A}}$ and $\mathrm{p18}^{\mathrm{INK4C}}$ immunohistochemistry on the GBM tissue microarray (TMA). iCys % refers to quantitation of cells with detectable immunoreactivity. Examination and grading by neuropathologist K.L.L. assigned Score and Intensity to each core. Final calls for expression (positive or negative) were based on neuropathologist reading. Parameters for the scores were as followed:

0 = 0% cells stain positive

1 = <10% of cells stain positive (scattered positive cells)

2 = 10-50% of cells stain positive

3 = 50-80% of cells stain positive

4 = >80% of cells stain positive

Table S5: Glioma cell lines in which *CDKN2C* (*p18*^{INK4C}) was sequenced.

Table S6: Sequences of primers used in competitive PCR.

References

Conrad, D. F., Andrews, T. D., Carter, N. P., Hurles, M. E., and Pritchard, J. K. (2006). A high-resolution survey of deletion polymorphism in the human genome. Nat Genet *38*, 75-81.

Furnari, F. B., Lin, H., Huang, H. S., and Cavenee, W. K. (1997). Growth suppression of glioma cells by PTEN requires a functional phosphatase catalytic domain. Proc Natl Acad Sci U S A *94*, 12479-12484.

Hinds, D. A., Kloek, A. P., Jen, M., Chen, X., and Frazer, K. A. (2006). Common deletions and SNPs are in linkage disequilibrium in the human genome. Nat Genet *38*, 82-85.

Iafrate, A. J., Feuk, L., Rivera, M. N., Listewnik, M. L., Donahoe, P. K., Qi, Y., Scherer, S. W., and Lee, C. (2004). Detection of large-scale variation in the human genome. Nat Genet *36*, 949-951.

Ishii, N., Maier, D., Merlo, A., Tada, M., Sawamura, Y., Diserens, A. C., and Van Meir, E. G. (1999). Frequent co-alterations of TP53, p16/CDKN2A, p14ARF, PTEN tumor suppressor genes in human glioma cell lines. Brain Pathol *9*, 469-479.

McCarroll, S. A., Hadnott, T. N., Perry, G. H., Sabeti, P. C., Zody, M. C., Barrett, J. C., Dallaire, S., Gabriel, S. B., Lee, C., Daly, M. J., and Altshuler, D. M. (2006). Common deletion polymorphisms in the human genome. Nat Genet *38*, 86-92.

Sebat, J., Lakshmi, B., Troge, J., Alexander, J., Young, J., Lundin, P., Maner, S., Massa, H., Walker, M., Chi, M., *et al.* (2004). Large-scale copy number polymorphism in the human genome. Science *305*, 525-528.

Sharp, A. J., Locke, D. P., McGrath, S. D., Cheng, Z., Bailey, J. A., Vallente, R. U., Pertz, L. M., Clark, R. A., Schwartz, S., Segraves, R., *et al.* (2005). Segmental duplications and copy-number variation in the human genome. Am J Hum Genet *77*, 78-88.

Tuzun, E., Sharp, A. J., Bailey, J. A., Kaul, R., Morrison, V. A., Pertz, L. M., Haugen, E., Hayden, H., Albertson, D., Pinkel, D., *et al.* (2005). Fine-scale structural variation of the human genome. Nat Genet *37*, 727-732.
